# The Bayes factor to discriminate Molière and Corneille as authors of classical French plays

**DOI:** 10.1038/s41598-025-24499-2

**Published:** 2025-11-19

**Authors:** Silvia Bozza, Valentina Cammarota, Claude-Alain Roten, Valentin Roten, Antoine Jover, Franco Taroni

**Affiliations:** 1 Department of Economics, Ca’ Foscari University of Venice, Venice, 30121 Italy; 2 OrphAnalytics SA, Vevey, 1800 Switzerland; 3 School of Criminal Justice, University of Lausanne, Lausanne, 1015 Switzerland

**Keywords:** Scientific data, Statistics

## Abstract

A question of originality was raised concerning the authorship of Molière’s plays. It has been claimed that the plays were written by Corneille in the final part of his life. This controversy is still topical, despite the relevant contributions on the subject (e.g.,^1^). Stylometry is often invoked to identify patterns used within and between words and sentences that describe a personal way of writing texts, and so one’s style. Despite the pioneering contribution of^2^, who promoted a Bayesian procedure for assessing the authorship of disputed documents, a coherent and judicially sound approach for features assessment and authorship attribution is still lacking in current practice. A full Bayesian framework to deal with the questioned authorship of the selected plays is promoted to address this controversy in total respect of (a) international standards for evaluative reporting in forensic science and (b) legal jurisprudence. Results strongly support the hypothesis that Corneille did not write Molière’s literary plays.

## Introduction

Jean-Baptiste Poquelin, known by his stage name as Molière, was a French playwright and poet. No date of birth is known, but it is reported that he was baptized on January 15, 1622. In 2022, we celebrated the $400^{th}$ anniversary of the baptism of this artist, who is widely regarded as one of the greatest writers in the French language and world literature. He is mainly known for his comedies; examples are *L’École des maris* (1661), *L’École des femmes* (1662), *Le Misanthrope* (1666), *Le Tartuffe* (1664) and *Les Femmes savantes* (1672), a list of plays that have been translated into every major living language. Other playwrights, such as Pierre Corneille (1606–1684), have not had the same public success with the lyrics of their comedies. From 1632 onwards, Corneille renewed his repertoire by composing tragedies; 23 plays of tragic inspiration characterized him and brought him great success with the critics and the public.

A question of originality was raised about Molière’s plays; some quarters critically claimed that the plays were written by Corneille in the final part of his life. It can be read - as reported in^3^ - that:

There is much evidence showing that Molière did not write his plays. In France, during the $17^{th}$ century, comedies were presented by comedians - like Molière - and not by the authors who wrote them. Molière did not behave as an author and none, among contemporaries, considered him a writer. In contrast, many claimed that Corneille wrote some of these plays. Those claims are confirmed by several statistical indices: intertextual distance, classifications, combinations of common words, meaning of keywords, sentence length. (at p. 117)

Corneille collaborated with Molière for the writing of the play *Psyché* (1671)^4^. During that period, a number of collaborations emerged between playwrights. Just using historical documents, it is sometimes not possible to understand the division of labour between authors. As specified by^1^, the extent of a collaboration is most of the time uncertain. The same authors in a later publication^4^ also referred to a list of critical aspects a scientist should take into account in an authorship analysis. They noticed that, in that period, ‘these plays are often very homogeneous, which makes it more difficult to properly attribute texts. In particular, similarities induced by the literary genre and sub genre can be as strong as similarities induced by authors’ idiolect.’ (at p. 378). Their claims were supported by scientific literature (see, e.g.^5^).^4^ also emphasized that ‘Imitation could even go to the point of piracy and plagiarism, such as attempting to steal another author’s play, or led to disputes about the true source of a story.’ (at p. 378). It was further not uncommon for some parts of plays to be borrowed from others.^1^ remarked that ‘All this contributes to make the potential stylistic variations between authors remarkably tenuous.’ (at p. 2/14). Despite these critical observations and the fact that such Molière controversy is still considered intricate to solve, the authors supported Molière’s original authorship of the plays. They wrote:

It shows that, from any viewpoint adopted, it is very unlikely that P. Corneille or his brother Thomas would have been Molière’s ghostwriters. As they were, after a century-old debate, the only option deemed plausible, these conclusions strongly substantiate the idea that Molière indeed wrote his own plays. (at p. 6/14)

They have also remarked that ‘In Molière’s case, only P. Corneille has ever been considered as a potential ghostwriter.’ (at p. 2/14).

Uncertainty about the authorship of plays can be investigated through stylometry. Everyone uses a personal way of writing, and documents can be represented as numerical vectors suitable for capturing relevant features that describe a style that evolves with time^6^. Based on repeated features statistics, stylometric analysis is the study of an author’s writing style to approach questions mainly related to textual authorship^7^. Among the wide variety of stylometric features that can be used (including, e.g., function of words), character *n*-grams - a sequence of *n* contiguous characters - are often considered a very selective feature for authorship description^8^. An overview of various stylometric features sets can be found in^9^.

The task of authorship attribution has a long history in forensic science. For example, the individualization of one’s handwriting represents one of the most important area of activity in the legal context. Unfortunately, it must be said that the procedure depends to a large extent on the analyses by the examiners, who rarely quantify relevant characteristics of the writing and assess them in a qualitative or unstructured manner. Several articles (since^10^ till^11^ and^12^) have highlighted this lack of a structured approach, calling into question the practice and the reliability of the field. Relevant discussions on these issues can be found in^13^ and^14^. Authorship attribution has recently gained greater attention due to new applications in several fields (e.g., humanities scholarship, copyright issues, forensic analysis) and to the availability of new computational methods that allow the analysis process to be partially automated to support the examiner. An overview of such computational methods can be found in^9^. Among these, statistical techniques based on multivariate analysis (including, e.g., principal component analysis or cluster analysis) have largely been applied. The reader can refer to^15^ for an extensive application of such multivariate methods to linguistic data.

However, the above-mentioned computational methods are essentially implemented in practice as exploratory techniques, characterized by an emphasis on graphical displays and the absence of a probabilistic model that would allow for statistical inference concerning the hypotheses of interest. Questioned texts are in fact attributed by seeing which author’s comparison documents are closest. As noticed by^1^, visual discrimination based on the distance between groups is a ‘disturbing [inferential] procedure’ because ‘Observing that a text is closer to Corneille than to Quinault [an alternative plays writer] does not mean it is written by Corneille.’ (at p. 1/14). Unfortunately, no coherent and legally sound criteria for inference on authorship are implemented in current practice and the question of interest - i.e. how likely is that Corneille is the author of a given play?—remains unsolved.

Machine learning (ML) techniques have more recently gained great popularity for authorship attribution (see, e.g.,^16^). These methods have the undoubted advantage of enabling dimensionality reduction and discrimination in both a supervised and un-supervised learning process. Though ML techniques excel in detecting potential similarities, outliers and suspicious activities and may therefore represent valuable tools for optimization in many scientific or industrial domains, it must be acknowledged that their implementation has also raised criticisms. A critical view of ML techniques can be found in^17^. Their implementation in forensic science has also been recently criticized by^18^ and^19^ by noting that ‘the persistence of source attribution claims [individualization or authorship] in forensic science literature is problematic. [...] Machine learning not only bears potential for misapplication for the purpose of forensic source attribution, but that such misapplications actually do occur.’.

The major criticism is the failure of these techniques to provide an answer to the problem of evaluation and interpretation of evidence in support of the hypotheses of legal interest. This does not diminish the importance of the dimensionality reduction that can be achieved by their implementation when dealing with a large number of variables. An output of legal interest cannot, however, be the result of data analysis on its own. The data-centrism of the standard ML scheme is insufficient to provide an output of legal interest^20^. The joint assessment of multiple sources of information, as declared by Swiss jurisprudence (see, e.g.^21^), is needed. The discrimination process among competing hypotheses require supplementary information to assign a value to the probability that a certain individual or group is the source of the available evidence. This means that the role of the scientist is limited by legal definition (see, e.g.,^22^ at p. 467 (par. 2223)): the expert’s findings should be focused to an evaluation of the evidence under a set of alternative hypotheses of interest, and should not address the question of whether the hypotheses are true or false. Moreover, deciding on a hypothesis requires combining the information coming from the expert with prior knowledge about the case. This means, as expressed by^18^, that ‘An exclusively data-driven scheme will produce outputs that fall short the fundamental ingredients that define forensic problems, namely value judgements (i.e., preferences among decision consequences) and prior probabilities referring to other, non-scientific evidence available in the instant case’ (p. 12). If, in their testimony, the scientist conflates the two, it will be difficult for the Court to assess the value of the evidence properly^23^ and for the opposition to confront the expert effectively^19^. Scientists can help by offering to ensure that authorities’ conclusions are scientifically sound and logically defensible^24^. Such conclusions would satisfy the right to a fair trial (Article 6 of The European Convention on Human Rights) as noticed by^25^. Such conclusions also avoid fallacious reasoning and errors in legal proceedings which could put defendants at risk. For all these reasons, the criticalities of ML techniques in forensic science applications have recently been reiterated by^26^.

Within the constraints imposed by case law and rationality, and taking advantage of forensic published recommendations (see, e.g., ENFSI guidelines^27–29^ and the more recent comment made by^30^) on the way to measure the probative value of available findings, the current paper promotes a Bayesian probabilistic methodology for detecting authorship. The main goal is to provide a suitable tool for quantifying the support that the output of stylometric analyses of selected plays provides in favor or against the hypotheses that are of relevance, say ‘[a given play] was written by Molière’ versus the alternative hypothesis that ‘[a given play] was written by Corneille in his last period of life (from 1655 onwards)’. The measure of this support is given in probabilistic terms through a coherent metric known as *Bayes factor* (BF)^31–35^. The choice of a Bayesian framework makes it possible to distinguish between the respective roles of the expert and the fact-finder in the evaluation of the evidence, while being scientifically (see, e.g.,^32,36^), legally (see, e.g.,^37–41^), and philosophically (see, e.g.,^42–45^) sound. An early implementation for authorship detection was proposed by^2^, who considered linguistic features such as frequencies of words to determine the probability of authorship. More recently, the use of the BF has been proposed by^46^ to discriminate between hypotheses on authorship of various multilingual texts written by humans or by intelligence media.

The paper provides a comprehensive discussion on this issue and is structured as follows. Section ‘Stylometry’ briefly defines stylometry as a methodology to deal with text analysis through lexical or grammatical features. Section ‘The Bayes factor for assessing the value of findings’ introduces the readers to the use of the Bayes factor as a measure for scientific findings evaluation and describe the statistical model that can be adopted to deal with available stylometric data. An extension to situations involving composite hypotheses is also discussed. Section ‘Materials and methods’ presents the available text materials and the methods used for the extraction of data from plays historically attributed to the retained authors. It is shown how the suggested probabilistic approach may offer a valuable contribution to help tackling the question of authorship. Results of performed analyses are presented in Section ‘Results’, where the impact of choices concerning, e.g., the selection of *n*-grams or the type of distance measures for the implemented multivariate analysis technique, is also studied. The inferential method can be extended to encompass a perspective of decision-making. This is illustrated in Section ‘A step further: a decision-making perspective’. Section ‘Conclusion’, finally, concludes the paper.

## Stylometry

A text consists of words structured by the turns of phrase (syntax) of an author^47^. The semantic signal, which reflects the significance and interpretation of words and phrases^48^, may be thought to be dominant in a text. However, the syntactic signal also largely predominates in a text because it characterizes the writing style of a writer^49^. If the authorship can be determined by semantic or syntactic methods, stylistic studies show that syntactic analyses are efficient to support an author’s identity^50^. This type of qualitative analysis, however, is time-consuming and requires the assistance of specialized expertise in the language of the text. Because of their speed, algorithmic approaches to text authorship differ from classical linguistic methods. The algorithms adopted in this contribution have been validated on the basis of genomic analyses and represent a robust approach for data collection; they allow the characterization of a document by means of the systematic identification of pattern. The word *genomics* refers to a description of a sequence of nucleotides DNA. Each nucleotide is composed of 1 of 4 nucleotides, the order of which determines the genetic sequence. Analogously, the order of a number *n* of contiguous characters (identified by the term *n*-gram^51^) in a text characterizes an author’s style. It identifies patterns used in and between words and sentences^6^. This algorithmic approach measures the most frequent signal in a text, the syntax, and thus the style of a text. The stylometric profile of texts of an alleged author allows one to rule quantitatively on such texts to tackle the problem of authorship using proximity measures with texts whose authorship is questioned^7,52^. A review of the literature on stylometry can be found in^53^.

Though this may represent a valuable item of information for investigation, it is essentially descriptive, and the lack of an inferential and decision-making approach is evident. The style markers (selected features or a function of them, such as a distance or a similarity score) can be used in association with a probabilistic approach for supporting authorship.

## The Bayes factor for assessing the value of findings

The evaluation of scientific evidence may be thought of as the assessment of measurements on relevant features taken from some material collected and analysed by experts or investigative authorities. However, it must be admitted that the reconstruction of facts is inevitably characterised by uncertainty, which cannot be eliminated but can be measured consistently through probability^54,55^. Probability and statistics play a key role in assessing the support provided by the evidence for the reconstruction of unknown scenarios (e.g., the authorship of a questioned document is uncertain). This central role of probability in the evaluation of scientific evidence is supported by institutions like the European Network of Forensic Science Institutes (ENFSI), which plays a coordinating role for forensic science in Europe. In the ENFSI Guidelines for evaluative reporting in forensic science published in 2015, it is indicated, at page 6 (under point 2.3), that:

Evaluation of forensic science findings in court uses probability as a measure of uncertainty. This is based upon the findings, associated data and expert knowledge, case specific propositions and conditioning information.

where the term ‘findings’ denotes evidence, observations or measurements in this context.

This ENFSI recommendation does not only explicitly support the use of probability to quantify uncertainty, but it also prescribes how to evaluate evidence, notably through a metric called *likelihood ratio* (or *Bayes factor* in a Bayesian framework):

Evaluation will follow the principles outlined in [...]. It is based on the assignment of a likelihood ratio. Reporting practice should conform to these logical principles. This framework for evaluative reporting applies to all forensic science disciplined. The likelihood ratio measures the strength of support the findings provide to discriminate between propositions of interest. It is scientifically accepted, providing a logically defensible way to deal with inferential reasoning. (point 2.4, at p. 6)

Recall the authorship question raised in the Introduction and denote by $H_1$ and $H_2$ the following hypotheses:

$$H_1$$:
The author of a given play [...] is Molière;
$$H_2$$:
The author of a given play [...] is Corneille in his last period of life (from 1655 onwards).

Denote by $\Pr (H_l\mid I)$, $l=1,2$, the probability representing prior degree of belief in hypothesis $H_l$, where *I* denotes the relevant information available at the time when the probability assessment is made. Suppose that a questioned document is divided into *n* parts and that stylometric analyses are performed. Denote the measurements on the questioned document by $\textbf{y}=(\textbf{y}_{1},\dots ,\textbf{y}_n)'$, where $\textbf{y}_i=(y_{i1},\dots ,y_{ik})'$, $i=1,\dots ,n$ and *k* represents the number of retained features. Prior probabilities $\Pr (H_l\mid I)$ and the probability of the evidence given the hypotheses, $f(\textbf{y}\mid H_l)$, can be combined by means of Bayes’ theorem to obtain the posterior probabilities of the hypotheses of interest, $\Pr (H_l\mid \textbf{y},I)$. A probabilistic method for discriminating among competing hypotheses was proposed by^31^ and is given by the so-called *Bayes factor* (BF for short):

1 $$\begin{aligned} \textrm{BF}=\underbrace{\frac{\Pr (H_1\mid \textbf{y},I)}{\Pr (H_2\mid \textbf{y}, I)}}_{\text {Posterior odds}}/\underbrace{\frac{\Pr (H_1\mid I)}{\Pr (H_2\mid I)}}_{\text {Prior odds}}=\frac{\Pr (H_1\mid I)f(\textbf{y}\mid H_1,I)}{\Pr (H_2\mid I)f(\textbf{y}\mid H_2,I)}/ \frac{\Pr (H_1\mid I)}{\Pr (H_2\mid I)} =\frac{f(\textbf{y}\mid H_{1},I)}{f(\textbf{y}\mid H_{2},I)}. \end{aligned}$$

The BF measures the change produced by the collected measurements in the odds in favour of $H_1$ when going from the prior odds to the posterior odds. Notice that the Bayes factor is a ratio of marginal likelihoods. It is greater than zero (except when $f(\textbf{y}\mid H_{1},I) = 0$ in which case it is equal to zero) but has no theoretical upper limit. This factor has intuitively pleasing properties. A value greater (less) than one in which the evidence has a higher (lower) probability if $H_{1}$ is true than if $H_{2}$ is true increases (decreases) the odds in favour of $H_{1}$. This means that values of the BF greater (less) than 1 support the hypothesis $H_1$ ($H_2$) at the numerator (denominator) of the ratio, and values equal to 1 are said to be ‘neutral’; it means that the findings are equally likely under the competing hypotheses and do not allow for discrimination between them.

It should be emphasised that a BF supporting a hypothesis does not indicate that the latter is more probable. The forensic scientist’s own task is to assess this value: it is then up to the judge (or jury) on the basis of available information, to quantify its uncertainty about the case and assess its prior odds. Scientists can inform recipients of expert information on how to change their prior odds in the light of the evidence, but scientists cannot by themselves assign a value to the prior or posterior odds. In order to assign such a value, all the other evidence in a case has to be considered, as underlined by law jurisprudences.

It is widely accepted that the forensic scientist must consider at least a couple of competing hypotheses, but there are circumstances in which the hypotheses of interest may even be more than two. For example, the hypothesis of interest could be formulated as follows:

$$H_{1}$$:
The author of a given play [...] is Molière;
$$H_{2}$$:
The author of a given play [...] is Corneille in his last period of life (after 1655); or
$$H_{3}$$:
The author of a given play [...] is Corneille in his first period of life (before 1655).

Corneille wrote both comedies and tragedies since 1655. Afterwards, he mainly wrote tragedies (note that just two comedies have been written after 1655, in 1670 and 1672, respectively). The Bayes factor to deal with multiple hypotheses takes the following form:

2 $$\begin{aligned} \textrm{BF}=\frac{f(\textbf{y}\mid H_{1},I) \sum _{l=2}^{m}\pi _l}{\sum _{l=2}^{m}f(\textbf{y}\mid H_{l},I)\pi _l}, \end{aligned}$$

where $m=3$ and $\pi _l=\Pr (H_l\mid I)$, $(l=1,\ldots ,m)$, represent the prior probabilities of the competing hypotheses such that $\sum _{l=1}^{m}\pi _l=1$. Note that the Bayes factor in Equation (2) is also a function of the prior inputs, $\pi _2$ and $\pi _3$, and not of the data alone^32^. Its interpretation is, however, unchanged.

## Materials and methods

### Experimental design

The question of authorship is approached through the analysis of a series of plays attributed to Molière and Corneille. It has to be noticed that in several instances in this paper, expressions such as ‘written by’ or ‘attributed to’ are used with reference to the authorship of a particular play. Note that no inference of authorship is made, and that it is only meant that a play has been historically attributed to a given author. Molière’s and Corneille’s syntax has been described using the following written material selected from editions available at https://theatre-classique.fr/ and https://obvil.sorbonne-universite.fr/corpus/moliere/:

- Molière: *L’Etourdi* (comedy, 1655), *Le Dépit amoureux* (comedy, 1656), *Sganarelle* (comedy, 1660), *L’Ecole des maris* (comedy, 1661), *L’Ecole des femmes* (comedy, 1662), *Le Tartuffe* (comedy, 1664), *Le Misanthrope* (comedy, 1666), and *Les Femmes savantes* (comedy, 1672);
- Corneille (before 1655): *Mélite* (comedy, 1625), *Clitandre* (comedy, 1632), *La Veuve* (comedy, 1632), *La Galerie du Palais* (comedy, 1633), *La Suivante* (comedy, 1634), *La Place Royale* (comedy, 1634), *Médée* (tragedy, 1635), *Horace* (tragedy, 1640) and *Le Menteur* (comedy, 1644);
- Corneille (after 1655): *Sophonisbe* (tragedy, 1663), *Othon* (tragedy, 1664), *Agésilas* (tragedy, 1666), *Attila* (tragedy, 1667), *Tite et Bérénice* (comedy, 1670) and *Pulchérie* (comedy, 1672).

A selection of plays that have been attributed to Molière, and a selection of plays that have been attributed to Corneille limited to those authored after 1655 are considered to deal with the authorship question of interest. Afterwards, also plays attributed to Corneille that are dated before 1655 are considered to approach situations involving composite hypotheses.

Each considered play was divided into several parts of equal character lengths in order to study their homogeneity. This makes it possible to operate on large texts. However, as this choice has an impact on overall performance, preliminary tests were conducted on such plays and the best performance was observed by splitting the analyzed manuscripts into five parts.

Stylometric analyses based on the observation of frequencies of selected character *n*-grams have been performed using the software PATOA developed by OrphAnalytics SA (www.orphanalytics.com). Character *n*-grams have been proved as being sensitive and accurate markers^56^ and effective in forensic contexts^9^. Previous studies have shown that the use of these markers is suitable for detecting different stylistic information, regardless of the language studied, provided that the texts to be compared were written in the same language^57^.^58^ conducted a study assessing the efficacy of 39 style markers. The use of 2-grams and 3-grams provided the best performance, although it turned out that longer *n*-grams are also effective indicators. This result could be explained by the fact that all the text analysed focused on the same topic. In fact, the longer the sequence of characters, the more words are detected. Thus, lexical elements, related to the theme of the text^7^, are considered. Although character-level *n*-grams do not represent clearly interpretable objects from a linguistic point of view^59^, they provide lexical, syntactic, and structural information^60,61^. The goodness of their use for linguistic purposes is also confirmed by^1^.^62^ presents a simple example using 3-grams: the article ‘the’ can provide lexical information, the inflection ‘-ing’, a syntactic item of information, and finally a structural information is obtained thanks to punctuation marks like ‘. T’ or ‘T.’.

**Fig. 1:**
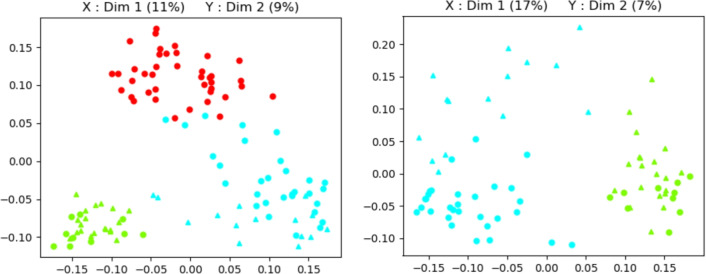
Biplot of the first two coordinate values of a principal coordinates analysis. Triangles and circles characterize tragedies and comedies; every triangle and circle represents a text section (out of 5) of one of the plays considered in this study. (top left) green symbols and red symbols characterize Corneille’s (after 1655) and Molière’s plays, respectively; (top right) green symbols, cyan symbols and red symbols characterize Corneille’s (after 1655), Corneille’s (before 1655) and Molière’s plays, respectively; (bottom left) cyan symbols and red symbols characterize Corneille’s (before 1655) and Molière’s plays, respectively; (bottom right) cyan symbols and green symbols characterize Corneille’s (before 1655) and Corneille’s (after 1655) plays, respectively.

Starting from the observed frequencies of the *n*-grams that are made available, a distance measure is chosen to quantify similarities between selected plays, and a principal coordinates analysis (PCoA) is conducted with the aim of reducing the dimensionality of data. This can be achieved by transforming the original proximity matrix, in such a way as to find a set of low-dimensional points that best approximates the original high-dimensional configuration with the least possible loss of information. These points are ordered so that the first ones represent the most variation in the original data. In this way, it is possible to use a small number of these new variables as a surrogate of the original large number of variables (see Fig. 1 (top left), where it is represented the output of such multivariate analysis for characterizing two different lists of plays). Note that two authors have been considered here, i.e., Molière and Corneille (in this latter case, only plays dated after 1655 have been retained). Figure 1 (top right) presents the output for three different lists of plays involving also plays dated before 1655 attributed to Corneille. Figure 1 (bottom left) presents the output of principal coordinate analyses for two different lists of plays involving Molière’s and the pre-1655 Corneille’s plays.

The influence of literary genre, which has been widely discussed and debated in the literature, has been investigated. According to^63^, an author can use different styles according to the text genre. As mentioned by^64^, there are stylistic features that depend on the subject of the text, and a literary genre for a given text. But, even in texts classified under different literary genres, some stylistic elements remain unchanged^65^ and the *n*-grams play a key role because they represent stylistic markers considered independent of the genre of the text. Molière’s plays are comedies. Ideally, given the hypotheses listed above, only Corneille’s comedies should be considered. Unfortunately, Corneille’s repertoire was mainly marked by tragedies in the time interval between 1655 and 1670, the same period in which Molière would have written his repertoire. The last comedies published after 1644 are in fact dated between 1670 and 1672. Since this is such a debated issue in the literature, we sought to understand to what extent the literary genre could actually influence the performed analyses. Figure 1 (bottom right) presents the output of principal coordinate analyses for different Corneille’s plays. By using only the plays attributed to Corneille, it is possible to investigate whether this proposal is suitable for discriminating between literary genres.

### Statistical analyses

Recall the main interest which primarily focuses on the discrimination between two competing hypotheses on the questioned authorship. The output of the PCoA is retained to create two databases, one for each author. The background data can be denoted by $\textbf{x}_{lij}=(x_{lij1},\dots ,x_{lijk})'$, where $l=1,2$ denotes the putative authors, $i=1,\dots ,m_l$ is the number of available texts whose source is known (i.e., formally attributed), $j=1,\dots ,n$ is the number of parts each text is divided into and *k* is the number of retained coordinate values. The first pair of coordinate values was retained (i.e., $k=2$). While this has the great advantage of reducing the dimensionality problem, it also - as can be seen in Fig. 1—allows good discrimination between the compared corpus and proves to be a good starting point for subsequent analyses. Note that a small/large distance does not automatically imply a high/low probability that the analysed texts were written by the same author. Distances (as well as other measures of similarity or dissimilarity) must be evaluated and the proposed statistical model serves precisely this purpose, i.e., to assign a value to the probability of obtaining such measure under the hypothesis that the questioned text was written by a given author or, alternatively, under the hypothesis that it was written by a different author.

The data structure suggests a two-level hierarchy, accounting for two sources of variation: the variation between measurements within the same text (the *within-source variation*), and the variation between different texts of the same author (the *between-source* variation). A Bayesian two-level random effects model can be proposed as in^66^. To illustrate the model, let us denote by $\varvec{\theta }_{li}$ the mean vector within-source *i* and by $W_l$ the within-source covariance matrix under hypothesis $H_l$, $l=1,2$. For the within-source variation, the distribution of $X_{lij}$ is taken to be Normal, $X_{lij}\sim N(\varvec{\theta }_{li},W_l)$. For the between sources variation, denote by $\varvec{\mu }_l$ the mean vector between sources, and by $B_l$ the between-sources covariance matrix. The distribution of $\varvec{\theta }_{li}$ is taken to be Normal, $\varvec{\theta }_{li}\sim N(\varvec{\mu }_l,B_l)$. This allows the marginal likelihood $f_l(\textbf{y}\mid H_l)$ to be obtained analytically

3 $$\begin{aligned} f(\textbf{y}\mid H_l)\propto \mid W_l\mid ^{-n/2}\mid B_l\mid ^{-1/2}\mid (nW_l^{-1}+B_l^{-1})^{-1}\mid ^{1/2} \exp \left\{ -\frac{1}{2}\left[ \text {tr}(S_lW_l^{-1})+ (\bar{\textbf{y}}_l-\varvec{\mu }_l)'\left( n^{-1}W_l+B_l\right) ^{-1}(\bar{\textbf{y}}_l-\varvec{\mu }_l)\right] \right\} , \end{aligned}$$

where $\bar{\textbf{y}}_l=\sum _{j=1}^{n} \textbf{y}_{lj}$, $S_l=\sum _{j=1}^{n}(\textbf{y}_{lj}-\bar{\textbf{y}}_l)(\textbf{y}_{lj}-\bar{\textbf{y}}_l)'$. Note that the letter *I* has been omitted for sake of simplicity.

Note that none of the performed tests for assessing multivariate Normality have shown strong evidence against Normality. However, it must be acknowledged that a multivariate Normal distribution may not always represent a reasonable assumption, and that different choices may be preferable, such as a kernel density distribution^66^.

The overall means $\varvec{\mu }_1$ and $\varvec{\mu }_2$, the within-source covariance matrices $W_1$ and $W_2$ and the between-source covariance matrices $B_1$ and $B_2$ can be estimated, for $l=1,2$, using the available background data as:

4 $$\begin{aligned} \widehat{\varvec{\mu }}_l= & \bar{\textbf{x}}_l=\frac{1}{m_l n}\sum _{i=1}^{m_l}\sum _{j=1}^{n} \textbf{x}_{lij};\end{aligned}$$

5 $$\begin{aligned} {{\widehat{W}}}_l= & \frac{1}{m_l(n-1)}\sum _{i=1}^{m_l}\sum _{j=1}^{n}(\textbf{x}_{lij}-\bar{\textbf{x}}_{li})(\textbf{x}_{lij}-\bar{\textbf{x}}_{li})';\end{aligned}$$

6 $$\begin{aligned} {{\widehat{B}}}_l= & \frac{1}{m_l-1}\sum _{i=1}^{m_l}n(\bar{\textbf{x}}_{li}-\bar{\textbf{x}}_l)(\bar{\textbf{x}}_{li}-\bar{\textbf{x}}_l). \end{aligned}$$

## Results

In this section, the performance of the proposed Bayesian probabilistic approach is illustrated and discussed. In particular, the attention is focused on the suitability of this approach to discriminate between two alleged authors (Subsection ‘The role of the author’), between literary genres (Subsection ‘The role of the literary genre’), and finally to discriminate in the presence of more than two alleged authors (Subsection ‘The role of multiple authors’).

### The role of the author

Consider, just for sake of illustration, the following case where the authorship of *L’École des femmes* is questioned. The propositions to be compared can therefore be formulated as follows:

$$H_{1a}$$:
The author of *L’École des femmes* is Molière;
$$H_{2a}$$:
The author of *L’École des femmes* is Corneille in his last period of life (from 1655 onwards).

The model parameters $\varvec{\mu }_1$, $W_1$ and $B_1$ characterizing the marginal likelihood in (3) at the numerator of the BF in (1), are estimated as in (4), (5) and (6) using the background data that is given by the *k* retained features obtained by applying principal coordinate analysis to the seven available remaining plays attributed to Molière (i.e., *L’Etourdi*, *Le Dépit amoureux*, *Sganarelle*, *L’Ecole des maris*, *Le Tartuffe*, *Le Misanthrope* and *Les Femmes savantes*).

Similarly, the model parameters $\varvec{\mu }_2$, $W_2$ and $B_2$ characterizing the marginal likelihood in (3) at the denominator of the BF in (1), are estimated as in (4), (5) and (6) using the background data that is given by the *k* retained features obtained by applying principal coordinate analysis to the six available plays attributed to Corneille in his last period of life (i.e., *Sophonisbe*, *Othon*, *Agésilas*, *Attila*, *Tite et Bérénice* and *Pulchérie*).

**Fig. 2:**
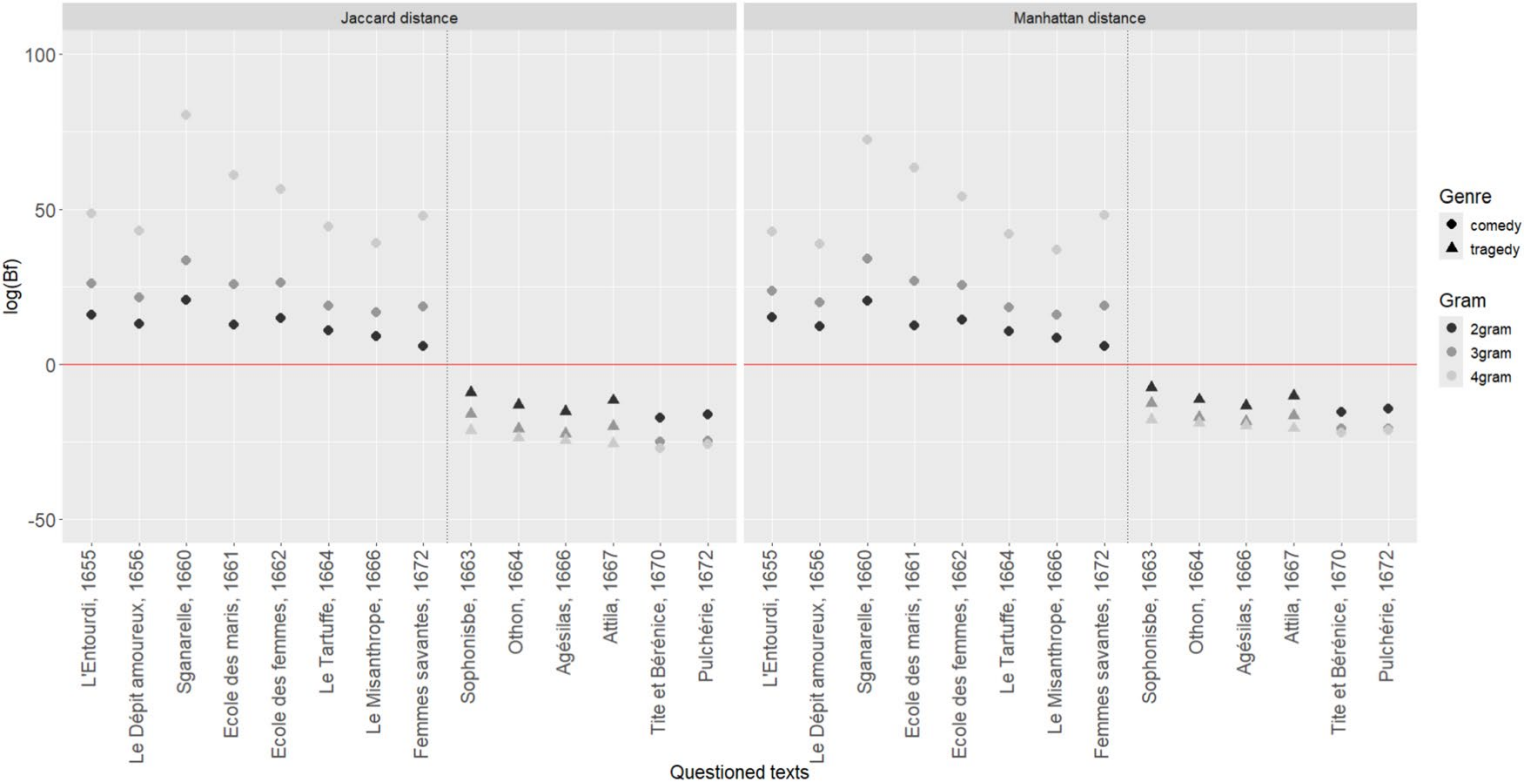
Weight of evidence, log(BF), as a function of the questioned plays (comedies: circle, and tragedy: triangle), the *n*-grams (black: 2-grams, grey: 3-grams, and light grey: 4-grams) and the distance measures (Jaccard and Manhattan). The vertical dotted line delimits the works bearing the authorship of Molière on the left and those of Corneille on the right.

The BF in (1) can be obtained as the ratio of (3) for $l=1,2$. If $H_1$ holds, a Bayes factor greater than 1 is expected. If $H_2$ holds, a Bayes factor less than 1 is expected. Several BF results have been obtained following a leave-one-out procedure, according to which a play is selected to act as evidence and all remaining plays are used as background data to estimate parameters. Results are presented in Fig. 2 where the logarithm of the Bayes factor, log(BF), is reported considering 2- to 4-grams as granularity using two alternative distance measures, the Jaccard and the Manhattan distances which are often quoted in scientific literature^58,67,68^.

Consider, for sake of illustration, the results obtained whenever the plays *L’École des femmes* and *Agésilas* are taken as questioned documents, in turn, 2-grams are retained and the Manhattan distance is chosen. Note that whenever *Agésilas* is taken as questioned document, the competing hypotheses are reformulated as follows:

$$H_{1b}$$:
The author of *Agésilas* is Molière;
$$H_{2b}$$:
The author of *Agésilas* is Corneille in his last period of life (from 1655 onwards).

The weight of the evidence^69^, measured by the logarithm of the Bayes factor (log(BF)), is equal to 14.4 and -13.3, respectively. The Bayes factors provide extremely strong support for both propositions: that the author of *L’École des femmes* is Molière (hypothesis $H_{1a}$), and the author of *Agésilas* is is Corneille (hypothesis $H_{2b}$). In accordance with the recommendations formalized by the ENFSI Guidelines^27^, such results provide extremely strongly support for the hypothesis that Molière authored *L’École des femmes* and for the hypothesis that Corneille authored *Agésilas*, respectively.

Is it therefore possible to conclude that Molière (or Corneille, in turn) most likely did write their plays? A note of clarification should be added. It should be emphasized that the scientist’s previous conclusions (explicited in terms of extremely strong support versus one of the competing propositions) explicitly refer to the observed data ($\textbf{y}$) conditional on the hypotheses (and all the choices made, e.g., the Bayesian statistical model adopted, the distance or the size of the *n*-grams) and not to the hypotheses conditional on the observed data and model assumptions. The difference is illustrated by the odds-form of Bayes’ theorem in (1). A logarithm of the BF of 14.4 indicates that, given the model assumptions, it is at least one billion times more probable that the measurements $\textbf{y}$ would have been observed if the author of *L’École des femmes* is Molière rather than Corneille in his last period of life (hypothesis $H_{1a}$). It is not the hypothesis that Molière authored a given play that is at least one billion times more probable. The value of the evidence (measured by the $\mathrm{BF}$) or the weight of evidence (measured by the $\mathrm{log(BF)}$) just supports one of the hypotheses regardless of how probable the hypothesis is. The confusion between the two conditional probabilities (i.e., between $\Pr (H_l\mid \textbf{y},I)$ and $f(\textbf{y}\mid H_l,I)$) characterizes what is known as the ‘transposed conditional’ error^70^, a pitfall of intuition also called ‘prosecutor’s fallacy’^71^ or ‘inversion fallacy’^72^. The ENFSI guideline for evaluative reporting describes the occurrence of such a pitfall by saying that ‘In the legal context, a fallacious transposed conditional statement is one that equates (or, confuses) the probability of particular findings given a proposition with the probability of that proposition given these findings.’ (at p. 27). Such a sentence reiterates what^73^ have pointed out to as a problem, which confirms our previous remarks about miscarriages of justice:

It is necessary for the scientist to consider the probability of the observations given each of the stated propositions. Not only it is not appropriate for the scientist to consider the probability of the proposition given the observations, there is a danger that in doing so the jury will be misled. (at p. 1)

It is fundamental that the scientist respects their role by offering to the recipient of the expert information the value of the evidence by calculating the Bayes factor or a function of it.

With reference to the results obtained, there is another aspect we wish to underline.^1^ noticed - by quoting past literature - that ‘Advocates of the thesis according to which P. Corneille would be Molière’s ghostwriter found that masterpieces in verse such as *Tarfuffe*, *Le Misanthrope*, or *Amphitryon* were the plays that raised the most suspicion.’ (at p. 6/14). The results obtained and displayed in Fig. 2, are however not in agreement with this hypothesis raised in the literature. However, it is worth noting that the weight of evidence for *Tarfuffe* and *Le Misanthrope* is weaker than that obtained for the other plays attributed to Molière and taken, in turn, as evidence, even if they continue to provide strong support for the hypothesis that Molière wrote these plays (in relation to the alternative that Corneille wrote the plays). The sole exception concerns *Les femmes savantes*, where the weight of evidence, if 2-grams are used, is even weaker, regardless of the distance measure used.

The BF values obtained using 4-grams are quite extreme, regardless of the type of distance used (Jaccard or Manhattan). This result is not surprising. The use of 4-grams is in-fact lexicon-related; this means that the context of the stories narrated in the plays and the thematics approached by the authors are factors that are capable of influencing the terminology used. Since the 4-grams consists of 4-character sequences, the probability of whole words of meaning being caught is higher compared to what can be observed whenever the length of the retained sequences of characters, thus the specific *n*-gram marker, is smaller than 4.

Extremely large values can be (theoretically) obtained (see, e.g., in Fig. 2 the BF values (in logarithmic scale) obtained whenever 4-grams are retained), and their meaning must be carefully investigated. Scientists, who have intimate knowledge of the assumptions behind the adopted statistical models and their appropriateness for the available data, should carefully take into account their limitations as well as their impact on conclusions.

Such a discussion is not new in forensic science, notably in the domain of DNA evidence where extremely tiny values for the occurrence of (conditional) genotyping profiles in given populations may be at the origin of huge values of the BF. Scientists are conscious that such large values invoke some statistical assumptions with a scale of robustness that cannot be demonstrated empirically given the size of currently available databases. Detailed discussions about such aspects dated back to^72,74^ and^75^ (see also^76^ and^77^ for a comment).

It is essential for a discrimination based on a Bayes factor that the method used to calculate the Bayes factor performs well. Several methods for the assessment of the performance of methods for the evaluation of evidence are available: in this article, we opted to measure the robustness of the Bayes factor by calculating the false negative and false positive rates. It can be noticed that numerical results presented in Fig. 2 showed no misleading evidence, that is cases where the Bayes factor points toward the wrong hypothesis. Recall that proposition $H_1$ has been formalized as ‘The author of a given questioned text [name of the questioned play] is Molière’, while proposition $H_2$ has been formalized as ‘The author of a given questioned text [name of the questioned play] is Corneille in his last period of life (from 1655 onwards).’ All Bayes factors obtained by choosing - in turn - each play as evidence supported the correct proposition: plays considered as written by Molière offered a Bayes factor greater than 1, or a log(BF)$>0$, while plays considered as written by Corneille gave values less than 1 or a log(BF)$<0$.

For a careful interpretation of the results, it is crucial that a scientist is able to answer these types of questions:

- How often might a scientist obtain a Bayes factor of this magnitude for plays that actually come from the Molière corpus (as assumed under hypothesis $H_1$)?
- How often might a scientist obtain a Bayes factor of this magnitude for plays that actually come from the Corneille corpus (as assumed under hypothesis $H_2$)?

Knowledge of the Bayes factor distribution under each of the competing hypotheses is necessary to answer these questions, regardless of the observed evidence. To empirically obtain such distributions and assess the overall ‘discriminating power’ of the chosen methodology, the impact of different choices in terms of *n*-grams, distances, or selected parts of the play must be investigated. The results, as illustrated in Fig. 2, show no errors.

Other methods for assessing a method’s performance are available, and we refer the reader to^32^, chapter 8 for a detailed discussion.

### The role of the literary genre

One might argue that plays of Molière and Corneille retained for this study are not of the same literary genre. Molière is known as an author of comedies, while the plays of Corneille, as a contemporary author of Molière, are essentially tragedies. For this reason, it was decided to investigate whether this aspect could influence the results. Seven comedies and eight tragedies, both authored by Corneille, were analysed. The competing propositions were formulated as follows:

$$H_{1}$$:
The Corneille’s play [...] is a comedy;
$$H_{2}$$:
The Corneille’s play [...] is a tragedy.

**Fig. 3:**
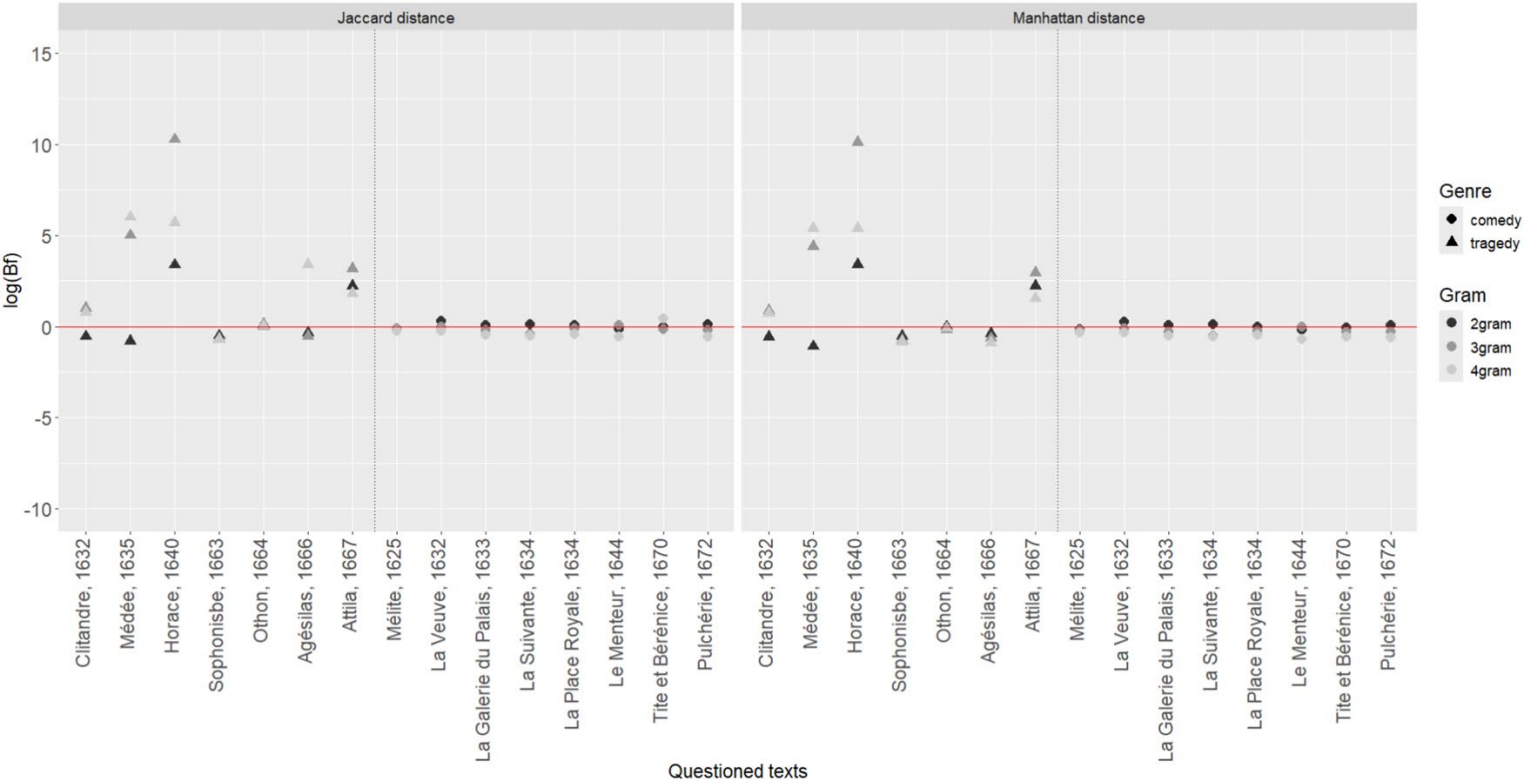
Weight of evidence (log(BF)) as a function of the questioned genre of plays selected among Corneille’s repertoire (commedy: circle, and tragedy: triangle), the *n*-grams (black: 2-grams, grey: 3-grams, and light grey: 4-grams) and the distance measures (Jaccard and Manhattan). The vertical dotted line delimits Corneille’s tragedies on the left and his comedies on the right.

Several BF results were obtained following, again, a leave-one-out procedure, according to which a play is selected to act as evidence and all remaining plays are used as background data to estimate model parameters. Results are reported in Fig. 3. One would expect a BF greater than 1 whenever the selected play is a comedy, and a BF less than 1 whenever the selected play is a tragedy. In contrast, the results obtained when comedies are selected as evidence and displayed in Fig. 3, tend to be *neutral* (close to 1, or to 0 in logarithmic scale) and suggest that the use of *n*-grams diminishes the effect of literary genre.

On the other side, BF values obtained whenever tragedies are selected as evidence are more sparse, but the list of plays considered in Section ‘Materials and methods’ suggest that Corneille’s tragedies are very close to Corneille’s comedies. These results support and justify the decision to use both comedies and tragedies to characterize Corneille’s style, in order to complete Corneille’s reference material with tragedies, since only two comedies after 1655 are available.

### The role of multiple authors

As already pointed out in Section ‘The Bayes factor for assessing the value of findings’, it could be of interest to compare more than two hypotheses. Let us therefore reconsider the same scenario as in Subsection ‘The role of the author’, where the questioned play was *L’École des femmes*, but the hypotheses to be compared are now formulated as follows:

$$H_{1}$$:
The author of *L’École des femmes* is Molière;
$$H_{2}$$:
The author of *L’École des femmes* is Corneille in his last period of life (after 1655); or
$$H_{3}$$:
The author of *L’École des femmes* is Corneille in his first period of life (before 1655).

Starting from equal prior probabilities for the competing propositions, $\Pr (H_1\mid I)=\Pr (H_2\mid I)=\Pr (H_3\mid I)=1/3$, and using the model parameter estimates obtained from the respective background data, it is possible to calculate the BF in (2). In particular, the weight of evidence is approximately equal to 3, a value that provides moderate support to the hypothesis that Molière wrote the play.

**Fig. 4:**
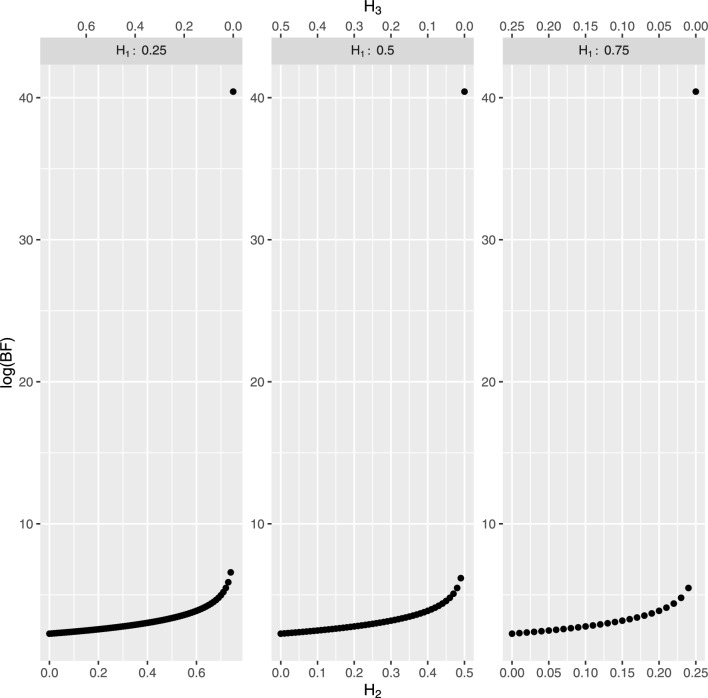
Weight of evidence (log(BF)) in favor of $H_1$ (the author of *L’École des femmes* is Molière) for values of $\Pr (H_1)$ equal to 0.25 (left), 0.5 (center) and 0.75 (right). The remaining probability mass is spread over hypothesis $H_2$ (the author of *L’École des femmes* is Corneille in his last period of life, see the *x*-axis on side 1) and hypothesis $H_3$ (the author of *L’École des femmes* is Corneille in his first period of life, see the *x*-axis on side 3).

The BF calculated in the presence of more than two hypotheses is also a function of their prior probabilities, as outlined in Section ‘The Bayes factor for assessing the value of findings’. In order to analyse the impact that different choices may have, a sensitivity analysis was conducted for values of $\Pr (H_1\mid I)$ equal to 0.25, 0.5 and 0.75, while the remaining probability mass was distributed over hypotheses $H_2$ and $H_3$. Results are shown in Fig. 4 for $\Pr (H_1)=0.25$ (left); for $\Pr (H_1)=0.5$ (center) and for $\Pr (H_1)=0.75$ (right). Values of $\Pr (H_2)$ and $\Pr (H_3)$ are displayed on side 1 and side 3 of the *x*-axis, respectively. If one sets the probability of hypothesis $H_3$ equal to 0 (i.e., one simply considers as an alternative hypothesis that Corneille in his last period of life (after 1655) was the author of the questioned play), the weight of evidence is approximately equal to 40. It is sufficient for the probability of hypothesis $H_3$ to be even slightly different from 0 to have a BF that offers less strong support to hypothesis $H_1$. If $\Pr (H_{2}\mid I)=0$, meaning that one just considers as alternative hypothesis that Corneille in his first period of life (before 1655) is the author of the questioned text, then the log(BF) equals to 2.26, no matter what is the probability of $H_1$. The fact that the values of the evidence are overall much lower than those observed when the alternative hypothesis was restricted to Corneille’s later period is not surprising. This finding aligns with the preliminary statistical analyses performed in the ’Material and methods’ section (see Fig. 1 (top right), where the first two coordinate values of the principal coordinate analysis clearly show that Corneille’s later writings are well-separated from Molière’s). However, it should be noted that, although with different strengths depending on the values of the a priori probabilities, the BF always points towards the hypothesis $H_1$. The same conclusions apply to any play historically attributed to Molière.

## A step further: a decision-making perspective

The Bayesian paradigm, according to which all uncertainties in a problem must be described by probabilities^78^, can be extended to encompass a perspective of decision-making. The issue of authorship attribution, discussed in this article, can in fact be formulated as a decision-making problem, i.e. deciding whether to attribute the authorship of a disputed play to one of the putative authors. To accomplish this task, the decision maker may rely on the one hand, to personal beliefs about the hypotheses of interest, and on the other, to personal assessments in terms of the undesirability of the consequences of each decision. Decision theory provides a unified framework to integrate all these aspects.

The combination of a particular decision with a particular hypothesis leads to a foreseeable consequence, to which one can associate a value that quantifies its undesirability and is called *loss*. Since it is unknown with certainty which hypothesis holds, one cannot directly say which decision leads to a consequence that is, in some sense, optimal. Each possible course of action, however, can be associated with a numerical value known as the *expected loss*, which is obtained by combining the losses associated with the decisions outcomes and the degrees of beliefs related to, say, two hypotheses:

$$\begin{aligned} \textrm{EL}(d_i) =\sum _{l=1}^{2} \textrm{L}(d_i,H_l)\Pr (H_l \mid \textbf{y},I), \end{aligned}$$

where $d_i$ represents the decision to attribute a play to the author specified by the hypothesis $H_l$, and $\textrm{L}(d_i,H_l)$ denotes the loss that is incurred whenever decision $d_i$ is taken and the hypothesis $H_l$ holds (note that a loss equal to 0 is incurred whenever the correct decision is taken, that is when $i=l$). The result is a qualifier for the appropriateness of particular decisions. A standard decision rule instructs one to select the decision with the lowest expected loss. This represents a coherent classification procedure since it minimizes the probability of misclassification (see, e.g.,^79–81^). It can be shown (see, e.g.,^34^) that whenever there are assessed equal prior probabilities for the hypotheses of interest, and a symmetric loss function is chosen (that is, it is felt that adverse decision outcomes are equally undesirable) this amounts to decide $d_1$ ($d_2$) whenever the BF is greater (less) than 1. Returning to the case at hand, and observing the results obtained and depicted in Fig. 2, one can clearly see how this decision criterion brings further confirmation in support of the previous conclusions drawn by^1^: ‘[O]ur analyses show a clear-cut separation between all the plays by Molière and [...] Corneille. This substantiates the claim that none of the plays signed by Molière were written by Corneille.’ (at p. 3/14).

The advantage of a full Bayesian framework, such as the one discussed in this paper, is that it can easily be adapted to take into account for different prior probability assessments or non-symmetrical assessments in terms of the undesirability of decision consequences. This means that the decision threshold is not necessarily equal to 1^34^. In the present case, the strong support provided by the evidence renders the impact of the different probability and loss assessments negligible, and the conclusions in terms of optimal decisions remain unchanged.

## Conclusion

A question of originality was raised about the authorship of plays historically attributed to Molière. At this regard, stylometric analyses may offer a valuable contribution to describe one’s style of writing. However, a coherent criterion for inference and decision on authorship is lacking in current practice. The aim of this paper is to promote a Bayesian probabilistic approach for quantifying the support that available evidence in the form of output of stylometric analyses can provide in favor or against hypotheses of authorship. The measure of this support is given in probabilistic terms by means of a Bayes factor, a coherent metric for evidence evaluation that is recommended by different forensic disciplines, but also by legal and philosophical literature^82^.

Bayes factor values strongly support the originality of Molière and Corneille plays, respectively, in full accordance with conclusions delivered by^1^. As it has been outlined in this paper, results are sensitive to multiple choices, included the type of stylistic features (i.e., *n*-grams), the plays used to implement the principal coordinates analyses, the number of sections in which a text is divided, the type of *n*-grams (i.e., the size of character sequences), the distance that is used, or the Bayesian probabilistic model that is adopted. A careful reading and interpretation of the results is therefore highly recommended, with a particular attention to the meaning and reasonableness of large BF values that can be obtained. Model validation measures^83^ are available to support the adoption of a model. The robustness of the Bayes factor did not indicate any errors in the value of the evidence, even with our limited, non-extensible datasets. Although large Bayes factor values might call for a better model (see^84,85^ and^32^), the proposed Bayesian model performs well for the specific task it was applied to.

It must be underlined that assessing the value of the BF (accompanied by an assessment of model performances) completes the forensic scientists’ task, but it is not the end of the matter. It is undeniable that very high values (i.e., in support of hypothesis $H_1$) or very small values (i.e., in support of hypothesis $H_2$), such as those observed here, can be quite compelling in one way or the other, but they cannot in themselves be decisive for the question at hand. A further step is required. The argument developed throughout this paper is that conceptual frameworks provide standards of reasoning useable to examine whether a given argument has the necessary credentials in order to be considered sound and, thus, whether reasoners are logically entitled to their conclusions. The Bayesian framework corresponds to a normative perspective for inference and decision which has the advantage of providing arguments in favor of the division of work between the scientist and the legal decision-maker, and of the clarification of the role of scientific evidence in a judicial context.

Finally, a last remark is necessary. Some might argue that the selection of plays by the two authors is numerically too limited and that a larger sample would be needed. However, it should be noted that forensic practice is usually confronted with a limited amount of trace material and comparative reference samples. An example of an application of such Bayesian framework under constraint of limited sample size was proposed by^46^. The historical scenario of interest concerns a specific copyright issue and in this case the material is largely abundant given the possibility of using the entire text of the various works. This situation is totally different from the scenarios usually encountered in forensic science, which are dictated by the scarcity of material both in terms of the questioned text and/or the reference material from various potential authors. Material availability is a recurrent problem and scientific conclusions have to deal with a careful analysis of such availability (see, e.g.,^56^). It is important to study the robustness of an evaluation metric - such as the Bayes factor - to the size of the background population. See, e.g.,^86^ and^87^ for handwriting analysis, and^88^ for signature analysis.

## Data Availability

The data underlying this article will be shared on reasonable request to the corresponding author.
